# Comparison of the Treatment Efficacy of Endo−Perio Lesions Using a Standard Treatment Protocol and Extended by Using a Diode Laser (940 nm)

**DOI:** 10.3390/jcm11030811

**Published:** 2022-02-03

**Authors:** Elżbieta Dembowska, Aleksandra Jaroń, Aleksandra Homik-Rodzińska, Ewa Gabrysz-Trybek, Joanna Bladowska, Grzegorz Trybek

**Affiliations:** 1 Department of Periodontology, Pomeranian Medical University, 70-111 Szczecin, Poland; elzbieta.dembowska@pum.edu.pl (E.D.); zperio@pum.edu.pl (A.H.-R.); 2 Department of Oral Surgery, Pomeranian Medical University in Szczecin, 70-111 Szczecin, Poland; jaronola@gmail.com; 3 Department of Diagnostic Imaging and Interventional Radiology, Pomeranian Medical University, 71-242 Szczecin, Poland; ewa_gabrysz@wp.pl; 4 Department of General and Interventional Radiology and Neuroradiology, Wroclaw Medical University, 50-369 Wrocław, Poland; Joanna.bladowska@umed.wroc.pl

**Keywords:** endo−perio lesions, diode laser, CBCT, periodontitis

## Abstract

Marginal and periapical periodontal diseases cause massive destruction of tooth tissues and surrounding tissues, such as alveolar bone and maxillary sinus floor, visible on radiographs. Lesions involving the apical and marginal periodontium are endo−perio (EPL) lesions. This study aimed to compare the treatment efficacy of endo−perio lesions using a standard treatment protocol and a standard diode laser-assisted treatment protocol. The 12 patients were divided into the study (a) and control (b) group. Periodontal indices, tooth vitality and mobility, occlusal status, and radiographic diagnosis were evaluated. Standard EPL treatment was then performed—without (a) and with (b) the use of diode laser (940 nm). Again, after six months, the above-mentioned parameters were evaluated and compared. The treatment of endo−perio lesions is a significant challenge for modern dentistry. Diode lasers are increasingly used in addition to traditional treatment methods. The conventional use of a 940 nm diode laser with an average power of 0.8 W in pulsed mode allows for the depth of periodontal pockets to be reduced. In addition, the use of a diode laser has a significant effect on tooth mobility and reduces bone loss.

## 1. Introduction

Marginal and periapical periodontal diseases cause massive destruction of the tooth’s tissues, and surrounding tissues, such as alveolar bone and the floor of the maxillary sinus, visible on radiographs [1]. Lesions that involve apical and marginal periodontium are endo−perio lesions (EPL). According to the 2017 classification, this type of lesion affects people with and without periodontal disease [2]. Advanced periodontitis results in the loss of connective tissues and increased depth of periodontal pockets. Secondary changes begin in the pulp of the tooth. Initially, the pulp is in a reversible inflammation state, but irreversible inflammation develops over time. The two are closely related structurally and functionally. Three main connection pathways are responsible for the occurrence of EPL: the main canals of the dental roots, the lateral and accessory canals, and the dentinal canals [2,3,4,5,6,7].

In 2017, a new classification of endo−periodontal lesions was formulated by Herrera et al. (Table 1). The authors divided the lesions into two groups: endo−periodontal lesions with root damage and without root damage. This new concept has changed the clinical approach, because the primary source, endodontic or periodontal, is not relevant to treatment. The diagnosis of an endo−periodontal lesion must answer whether to preserve the tooth or remove it. In the evaluation, there are three types of diagnosed EPL tooth: hopeless, which is classified for removal; bad; or favorable, which should be cured [2].

Treatment of endo−periodontal lesions involves the elimination of pathogens found in periodontal pockets and infected root canals [2,3,8]. The bacteria that are found in both of these environments are very similar. This similarity between bacteria is related to specific conditions, and the occurrence in anaerobic environments [9]. Some studies have shown that most bacteria are located in the outer 300 μm of dentinal tubules. These sites may be reservoirs from which the bacterial recolonization of treated root surfaces can arise [10]. Pathogens found in these types of lesions include *Streptococcus*, *Peptostreptococcus*, *Eubacterium*, *Bacteroides*, and *Fusobacterium*. A study published in 2020 showed that endo−perio lesions can be observed in the endodontium and periodontium, mainly *Tannerella forsythia*, *Porphyromonas gingivalis*, and *Aggregatibacter actinomycetemcomintans* [5].

Treatment in EPL should be two-pronged—periodontal and endodontic. These approaches are mechanical non-surgical periodontal treatment, consisting of surface root planning (SRP) or SRD (surface root debridement). The area, after being cleaned, is prepared to receive the new adhesion. Periodontal surface and root debridement can be performed using hand instruments and ultrasonic scalars. A comparison of manual and ultrasonic instrumentation use indicates that it is not statistically significant [11,12].

The goal of the endodontic algorithm is to eliminate bacteria that are present in the root canals [13,14]. Many proposals and protocols for decontamination and root canal preparation with laser devices have been presented in the literature. The first conventional protocols are CLE (conventional laser endodontics), aPAD (antimicrobial photo-activated disinfection), and LAI (laser-activated irrigation). In recent years, erbium lasers operating with short SSP pulses, low power, PIPS (photon-induced photoacoustic streaming), and SWEEPS (shock wave enhanced emission photoacoustic streaming) have been introduced to endodontic treatment. Treatment is performed using special tips with tailpieces in the presence of irrigation solutions: 17% sodium edetate (EDTA) and 5.25% sodium hypochlorite (NaOCl). They were proven to be very effective, without causing thermal effects on dental hard tissues [15]. The mechanism of their antibacterial action is primarily due to the thermal effects of radiation. Due to the different wavelengths emitted, diode lasers differ in their absorption range in water, which affects the penetration depth of the radiation into the tubules, from 400 to 1000 μm [16,17].

The laser therapy in periodontal pockets is able to eradicate pathogens and avoid surgical treatment.

The above considerations inspired the authors to address this topic.

This study aimed to compare the effectiveness of treating endo−perio lesions using a standard treatment protocol and a standard treatment protocol augmented with a diode laser. Null hypothesis—there is no difference in the efficacy of treating endo−perio lesions using a standard treatment protocol and a standard treatment protocol augmented with a diode laser.

## 2. Materials and Methods

This study was designed as a randomized and controlled 6-month clinical trial. The study protocol was approved by the Ethics Commission of the Medical University (no. KB-0012/29/17) and was conducted in full accordance with ethical principles, including the WHO Helsinki Declaration (2008 version).

### 2.1. Subject Selection

Sixteen patients of the Department of Periodontology, Medical University, with endo−perio lesions were enrolled in the pilot study. All patients were diagnosed with stage III periodontitis [18]. Each subject gave informed consent after explaining the study protocol, risks, and benefits. Two patients in both groups were excluded from the study due to missed appointments. Both groups had the same number of teeth—six molars in the study group (G1) and the control group (G2). Seven men and five women aged between 35 and 58 years (mean ± SD 46.5 ± 11.5) participated in the study. The inclusion and exclusion criteria for the study are shown in Table 2.

**Figure 1:**
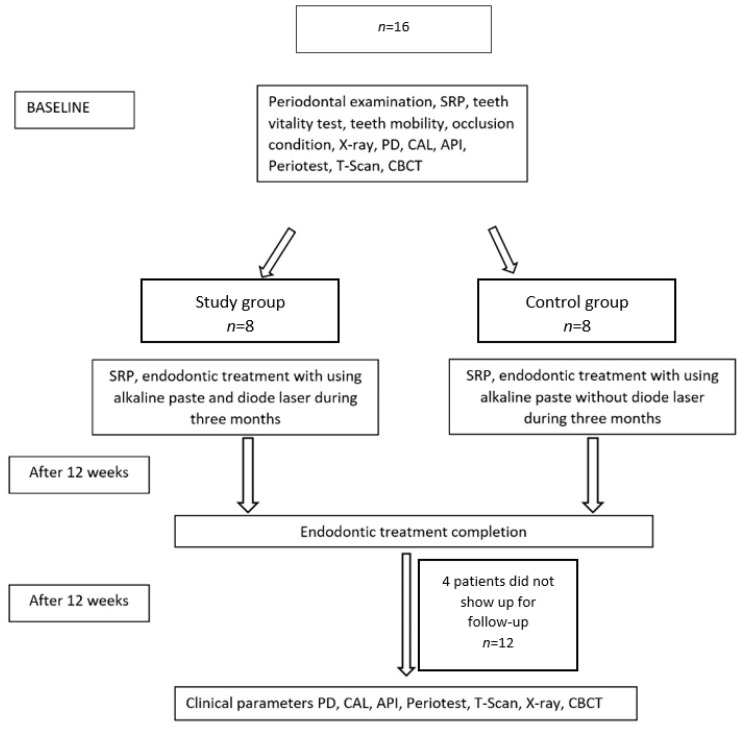
Study scheme.

The study was conducted according to the computerized random assignment of teeth to either the control (*n* = 6) or study (*n* = 6) group. Periodontal and endodontic treatment was performed in the first group, G1, with the additional use of a diode laser with a wavelength of 940 nm. The same procedures were performed in the control group G2, but without using the diode laser (Figure 1).

#### 2.1.1. First Visit

At the first visit, all patients (*n* = 12) were clinically examined for parameters such as periodontal pocket depth (PPD), tooth mobility, vitality test, occlusal status, and X-ray analysis. Periodontal pocket depth (PPD) was examined using a handheld periodontal probe (UNC 15, Hu-Friedy^®^, Chicago, IL, USA) at six sites. Classified teeth diagnosed with EPP showed no viability when tested with faradic current (PEm-1-type pulpoendometer) and ethyl chloride [19]. In addition, the study teeth were checked with Periotest M (Medizintechnik Gulden^®^, Modautal, Germany) in both groups before and after treatment. Periotest M is an instrument used to measure tooth mobility [20]. Every patient was checked using the T-scan Novus (Tekscan^®^, Boston, MA, USA).

In addition, before endodontic and periodontal treatment, cone bean computed tomography (CBCT) was performed to visualize bone defects in the vicinity of the tooth and to gain better insight into the anatomy of the root canal system of the treated tooth.

#### 2.1.2. Treatment

**Figure 2:**
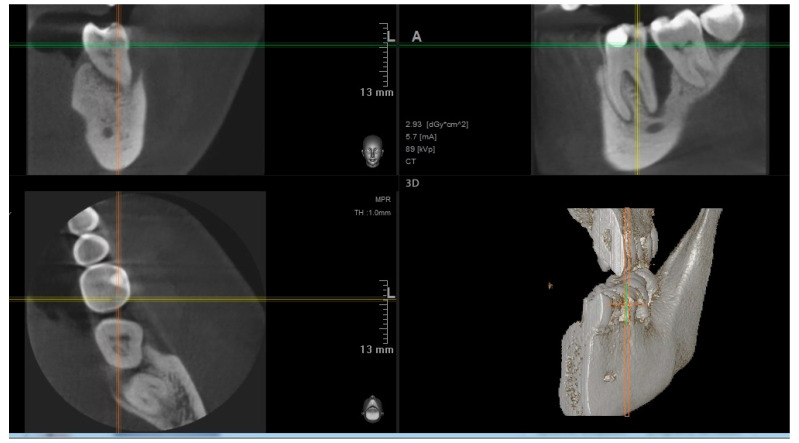
Baseline CBCT.

**Figure 3:**
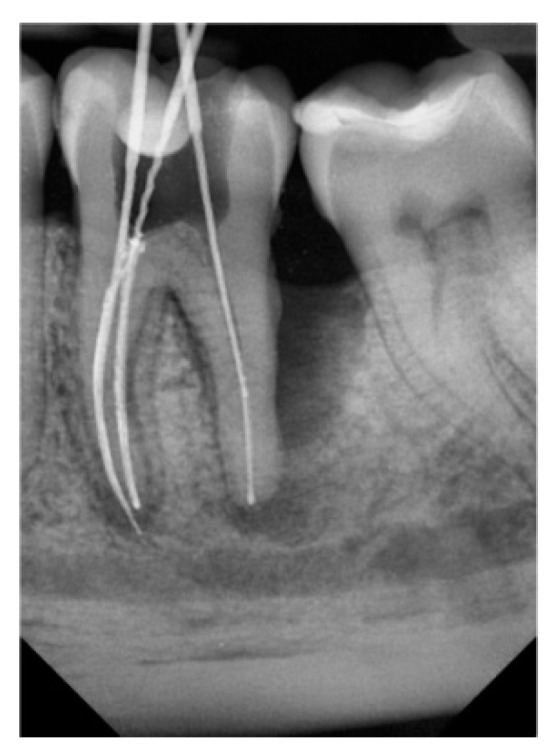
2D image during endodontic treatment.

Based on the examination, endodontic and periodontal treatment of the teeth was decided. Scaling and root planning of the teeth were performed using an ultrasonic scaler, and hand curettes were used. Intraoral radiographs (Pax Flex3D, Hwaseong-si, Gyeonggi-do, 18449, Korea) were then taken to classify the teeth, and endodontic treatment was started under anesthesia. The anatomy of the root system was evaluated on CBCT images before endodontic treatment (Figure 2), and endodontic treatment was performed using a microscope (Leica^®^, Wetzlar, Germany). A two-dimensional image was taken with the instruments in the canals (Figure 3). It was helpful to determine the working lengths of the canals, which were confirmed using a Raypex 5 endometer (VDW^®^, München, Germany). The rotary preparation was preceded by manually preparing the glide path using stainless steel hand instruments for a #20 file. The canals were prepared with 2% sodium hypochlorite, EDTA, and distilled water, using the crown-down method with the Endostar E3 Basic rotary system (Endostar^®^, Warszawa, Poland).

After complete canal preparation, the Epic X diode laser (Biolase^®^, Foothill Ranch, CA, USA) at 940 nm was used in group one. Disinfection was performed with sodium hypochlorite and the diode laser in a pulsed wave, with an operation input of 20 ms and output of 20 ms, average power of 0.8 W, pulse width of 20 ms, and timer of 10 s. The fiber of the diode laser tip with a diameter of 0.2 mm was introduced, which was shorter by 2 mm than the working length of the canals. There were three repetitions of disinfection per channel, with 10-s pauses between the repetitions. Calcium hydroxide paste Calcipast (Cerkamed^®^, Stalowa Wola, Poland) was applied to the canals between visits, and the teeth were temporarily sealed with Ketac Fil glass-ionomer cement (3M ESPE^®^, Maplewood, MN, USA).Root debritment was performed in each periodontal pocket. An EPIC X 940 nm laser (Biolase^®^, Foothill Ranch, CA, USA) with inactive tips was used to disinfect the interdental pockets without prior rinsing of the pockets. Pulse operation was completed with an input of 20 ms and output of 20 ms, average power per pulse of 0.8 W, and pulse energy of 32 mJ. The power density was 1132 W/cm^2^. The disinfection procedure used 300 µm diameter inactive quartz tips (E3-7). Each interproximal site was disinfected every 10 s. The fiber was inserted to the full depth of the pockets, for three repetitions in each pocket, with 10-s intervals between [8,21,22,23]. Disinfection of the pockets and canals was performed twice a month for three months. During all laser operations in the canals and periodontal pockets, the fiber was in continuous motion at a speed of 2 mm/s. After three months, it was decided to fill the canals with gutta-percha cones (Gutta Percha Points, Endostar^®^, Warszawa, Polska) with an AH Plus sealer (Denstply^®^, Charlotte, NC, USA) by lateral condensation. All of the laser parameters are shown in Table 3.

The same steps were performed in the control group, but without the diode laser.

After three months, the root canals were filled with gutta-percha cones (Gutta Percha Points, Endostar^®^, Warszawa, Polad) with AH Plus sealer (Denstply^®^, Charlotte, NC, USA).

After another three months, periodontal parameters, mobility, and CBCT were performed in both groups. Three-dimensional images were processed into special models to check the change of EPL volume.

**Figure 4:**
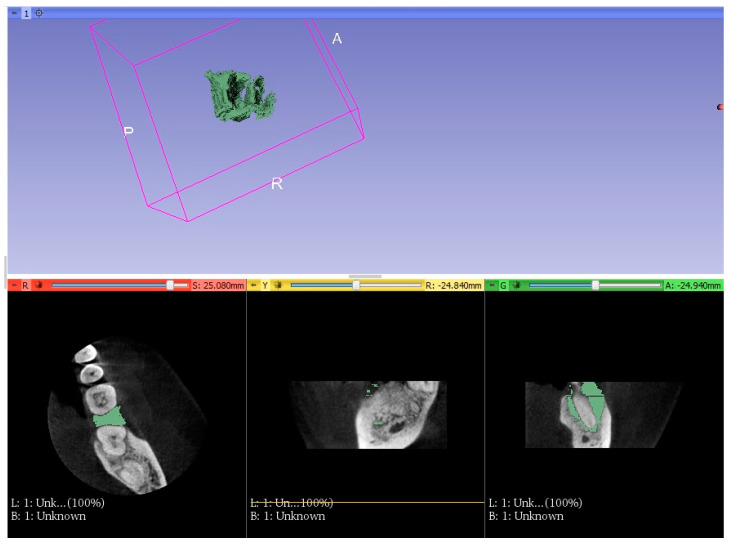
STL model before treatment.

**Figure 5:**
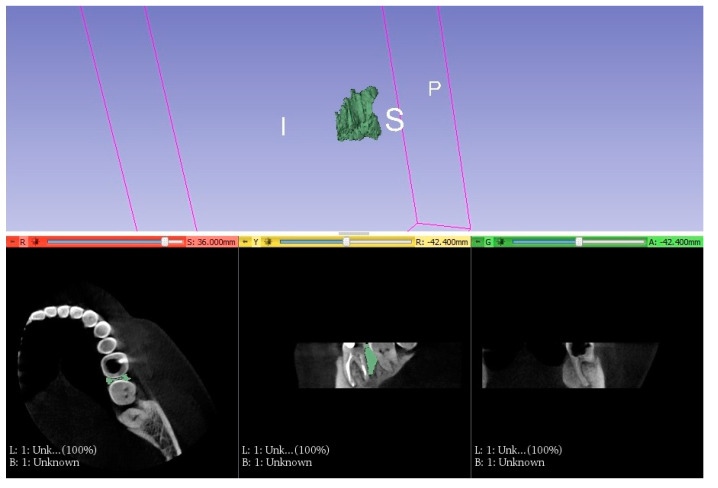
STL model after treatment.

Using CBCT, which was taken before and after treatment, STL (stereolithographic) models were made [14,15]. These models depicted the shape of the bone defects (Figure 4 and Figure 5). The effects of the completed treatment were also evaluated.

The CT scan was manually segmented in 3D Slicer software to visualize the lesion volume and bone defect regeneration. The files that resulted from the segmentation process were imported into Mini Magics 2.0 software to measure the bone defect volume [24,25,26].

### 2.2. Statistical Analysis

The STATA program (Version 15) was used for the statistical analysis. Pocket depth, mobility, and bone volume change were compared between the study and control groups. The significance of differences in the study and control groups at baseline and after treatment (after six months) was determined. Differences were considered statistically significant when the *p*-value was less than 0.05, and a trend at the limit of statistical significance was found when the *p*-value was 0.051–0.099.

## 3. Results

Baseline Characteristics

Statistical analysis showed a similarity in treatment initiation between the control and study groups. No statistical differences were found in terms of gender, age, and number of teeth (Table 4).

There were no significant statistical differences between the study and control groups in the pre-treatment examination, evaluating the six sites measuring the pocket depth. The groups were similar to each other. Before treatment, the pocket depth in the study group averaged 6.1 mm, and the deepest pocket was 13 mm (Table 5).

After treatment, the mean value of pocket depth was 4.22 mm, and the deepest pocket was 10 mm (Table 6). In the control group, the mean PD value was initially 6.03 mm, and the deepest pocket was 12 mm (Table 5). After treatment, the mean PD was 5.80 mm, and the deepest pocket was 10.5 mm (Table 6). The mean difference in PD was 1.88 mm in the treatment group and 0.23 mm in the control group. Significant statistical differences were found by comparing the measurements for all pocket depth sites in the study group before and after treatment. The Student’s test and Wilcoxon test were performed. However, significant statistical differences were found in the control group in only one pocket depth site. Due to the significant reduction in pocket depth in the study group, a significant difference was also found in the mean pocket depth values between the study and control groups (Table 7).

The mean value of Periotest in the study group before treatment was +14.08, and after treatment was +7.87 (Table 8). In the control group, the mean value before treatment was +14.77, and after treatment was +11.42 (Table 9). These results indicate that in the study group, the teeth decreased the mean mobility from 1°to 0°, which means physiological mobility. There was no statistically significant difference between the measurements before treatment in both groups during the statistical analysis. In the second measurement, there was a trend at the limit of statistical significance between the two groups in the Mann−Whitney test (Table 10).

As imaged by the STL models, the measurement of bone loss was performed in both groups before treatment, and no statistical difference was found in both groups. After treatment, checking the STL models in the study group, the lesions decreased by an average of 52.5% (Table 11), and in the control group, the lesions decreased by 27% (Table 12).

There was a statistically significant difference between the two groups after treatment between the study and control groups’ results. This was shown by the Mann−Whitney test and Student’s *t*-test (Table 13).

## 4. Discussion

The use of a diode laser is becoming increasingly popular in periodontal treatment. SRP is often assisted by laser therapy and is very effective for treating periodontal pockets, removing bacteria, and eliminating inflammation [27]. Lasers reduce the depth of periodontal pockets colonized by anaerobic bacteria, responsible for bone loss. This reduces bone lysis and improves tooth retention. The laser-activated irrigation (LAI) method is a straightforward protocol for rinsing and disinfecting the root canal. A fiber optic applicator with a diameter of 200–400 μ is placed approximately 4 mm from the apex. The procedure is performed in successive canals, rinsing is performed only at the end of the applicator, a minimal amount of rinsing solution is used, and the laser energy is directed directly into the dentinal tubules [21,28,29]. Moving the fiber minimizes the risk of thermal complications and allows the radiation to reach the lateral canal surfaces. The combination of diode laser, 5.25% NaOCl solution, and 17% EDTA solution allows for 100% elimination of *Enterococcus faecalis* [15,22]. Diode lasers provide effective removal of bacteria and toxins. In addition to bactericidal and detoxifying effects, diode lasers can accelerate wound healing, facilitate collagen synthesis, accelerate angiogenesis, and enable hemostasis [30,31]. Diode lasers are highly effective at removing the epithelium using a thermal mechanism [23,32]. Most studies on the efficacy of a diode laser as SRP and endodontic treatment support traditional methods with bactericidal effects, soft tissue debridement, and photobiomodulation [27,33,34,35,36]. Increasingly, the 940 nm diode laser is being used in dental practice to optimize periodontal and endodontic treatment efficacy. The 940 nm laser has an affinity for hemoglobin and melanin molecules. Its effectiveness is higher due to the fiber’s access to furcation areas, deep pockets, and root cavities [30,31]. It should be noted that the combination treatment is actually more effective in decontaminating the periodontal pocket, and it can also be assumed that recolonization is slower. The diode laser removes the mucosal epithelium more precisely than traditional hand tools, while the underlying connective tissue remains intact. According to some authors, lasers do not have apparent therapeutic effects [27]. Another work, a meta-analysis by Quadri [33], indicates that lasers give better therapeutic outcomes. The results of clinical studies on the use of diode lasers as an adjunct to the SRP procedure vary in the choice of parameters, e.g., wavelength, and the power ranges from 0.84, 1, 2, to 2.5 W in CW or pulsed mode. In some studies, the treatments were performed once or several times in a sequence of treatments. For this reason, the results reported in the available clinical studies on periodontal pocket treatment are difficult to compare and analyze [7,8,22,35,36,37]. In our study, we found a statistically significant reduction in pocket depth in the study group. In addition, diode lasers are beginning to be used as an aid in endodontic treatment for root canal disinfection. This is very helpful for reaching small dentinal canals and removing the smear layer. Diode lasers are available in a broad spectrum of wavelengths from 800 to 1064 nm, differing in their absorption properties. The 940 nm laser has an affinity for hemoglobin and melanin molecules. In addition, the benefits of diode laser and traditional SRP procedures in the treatment algorithm are associated with more significant bactericidal activity, a curettage effect, and a bio stimulatory effect. It should be noted that the combined treatment is actually more effective in decontaminating the pocket, and it can also be assumed that recolonization is slower [38]. Cone-beam computed tomography is also increasingly used in daily practice. The three-dimensional image is more precise than the two-dimensional image, and allows for more than one imaging layer to be visualized. A problem with the use of CBCT can be inexperience, thus incorrectly reading the image related to the artifact and gray tones [39,40]. This observation supports the use of traditional treatment methods with diode laser support in EPP to increase the efficiency of tissue regeneration and thus tooth maintenance, stopping the development of periodontitis. These processes should continue to be observed in 3D images.

This study has some limitations. Unfortunately, because this is a pilot study, the study group was not large. Due to the promising results obtained in our research, we plan to continue this study. Unfortunately, another limitation was the failure to conduct sample size calculations before beginning the study. The authors intend to conduct such a study in the future on a larger group, counting the power of the study and sample size calculations. Three-dimensional assessment of bone atrophy in treated teeth was evaluated in terms of its volume. For a more precise evaluation in further studies, we plan to compare the three-dimensional meshes obtained from STL files, their detailed evaluation in each dimension, and to determine the treatment effect on horizontal and vertical atrophy of alveolar bone [41].

## 5. Conclusions

The treatment of endo−perio lesions is a significant challenge for modern dentistry. In addition to traditional treatment methods, diode lasers are increasingly being used. The additional use of a 940 nm diode laser with an average power of 0.8 W in pulsed mode reduces periodontal pocket depth. In addition, the use of a diode laser has a significant impact on tooth mobility and reduces bone loss.

## Figures and Tables

**Table 1:** Endo−perio lesion (EPL) according to Herrera et al. [2].

Endo−periodontal lesion with root damage	Root fracture or cracking	
	Root canal or pulp chamber perforation	
	External root resorption	
Endo−periodontal lesion without root damage	Endo−periodontal lesion in periodontitis patients	Grade 1—narrow deep periodontal pocket in 1 tooth surface
		Grade 2—wide deep periodontal pocket in 1 tooth surface
		Grade 3—deep periodontal pockets in more than 1 tooth surface
	Endo−periodontal lesion in non-periodontitis patients	Grade 1—narrow deep periodontal pocket in 1 tooth surface
		Grade 2—wide deep periodontal pocket in 1 tooth surface
		Grade 3—deep periodontal pockets in more than 1 tooth surface

**Table 2:** Inclusion and exclusion criteria for enrolled patients.

Inclusion Criteria	Exclusion Criteria
Patients diagnosed with periodontitis, Grade III periodontitis Presence of endo−periodontal lesions without root damage Presence of at least 20 teeth Patients without increased tooth mobility Patients without occlusal problems or after occlusion correction Motivated patients with good oral hygiene (API < 15%)	Presence of systemic disease Patients taking antibiotics or immunosuppressive drugs six months before the study Pregnancy or lactation Smoking or alcoholism

**Table 3:** Laser parameters used during disinfection in periodontal pockets and root canals.

Localization	Fiber	Tip-Spot cm^2^	Pulse Width	% on Time	Average/Pulse Power W	Peak Power Dentisity W/cm^2^	Average Power Dentisity W/cm^2^	Total Energy mJ
Periodontal pocket (10 s)	300 μm	0.0007	input 20 ms/output 20 ms	50%	0.8/1.6	2264	1132	8
Root canal	200 μm	0.0003	input 20 ms/output 20 ms	50%	0.8/1.6	5093	2546	8

**Table 4:** Study group characteristics.

Sex	Group 1 (G1)	(%)	Group 2 (G2)	(%)	Summary
1 (MALE)	4	66.67%	3	50.00%	7
2 (FEMALE)	2	33.33%	3	50.00%	5
SUMMARY	6		6		12
Chi^2 Pearsons	0.34		df = 1		*p = 0.55819*
Fisher’s exact					*p = 1.0000*
R rang Spearman	0.17		t = 0.54233		*p = 0.59947*
NUMBER OF MOLAR	G1		G2		SUMMARY
16	0	0.00%	1	16.67%	1
27	1	16.67%	1	16.67%	2
36	4	66.67%	1	16.67%	5
37	1	16.67%	1	16.67%	2
46	0	0.00%	1	16.67%	1
47	0	0.00%	1	16.67%	1
SUMMARY	6		6		12
Chi^2 Pearson	4.80		df = 5		*p = 0.44078*
R rang Spearman	0.15		t = 0.48224		*p = 0.64001*

**Table 5:** Periodontal pocket depths (mm) before (B) and after (A) treatment in the study group (G1) around classified teeth.

Localization	Bucc. Mes. B/A (mm)		Bucc. Mid. B/A (mm)		Bucc. Dis. B/A (mm)		Ling. Mes. B/A (mm)		Ling. Mid. B/A (mm)		Ling. Dis. B/A (mm)		AVG.	AVG. Diff. (mm)
Patient													B/A (mm)	
1	5	4	5	3	10	7	4	3	4	3.5	13	10	6.1/4.25	1.88
2	4	3	6	2	7	3.5	4	3	5	4	5	4		
3	5	4	5	3	4	3	5	3	5	2	5	4		
4	8	4.5	6	4.5	6	4	7	6	5	4.5	5	4		
5	11	5	8	8	6	4	8	6	5	4.5	9.5	5		
6	6	5	6	3.5	5	3.5	6	4.5	6	4	6	4		

Bucc.—buccal; Ling.—lingual; Mes.—mesial; Dis.—distal; Mid.—middle; AVG.—average; Diff.—difference.

**Table 6:** Periodontal pocket depths before (B) and after (A) treatment in the control group (G2) around classified teeth.

Localization	Bucc. Mes.		Bucc. Mid.		Bucc. Dis.		Ling. Mes.		Ling. Mid.		Ling. Dis.		AVG. B/A (mm)	AVG. Diff. (mm)
Patient	B/A (mm)		B/A (mm)		B/A (mm)		B/A (mm)		B/A (mm)		B/A (mm)			
1	5	5	6	5.5	7	7	5.5	5	6	6	8	7.5	6.03/5.77	0.26
2	4	4	4	4	6	5.5	4.5	4.5	5	5	6.5	6.5		
3	5.5	5	8	8	7	6.5	6	5.5	6	5.5	7.5	7		
4	5	5	6	6	5.5	5.5	4.5	4.5	5	5	6	5.5		
5	12	10.5	3.5	3.5	6	5.5	7.5	7	4	4	5	5		
6	8	8	6.5	6.5	7	7	7	6.5	5	5	6	5.5		

Bucc.—buccal; Ling.—lingual; Mes.—mesial; Dis.—distal; Mid.—middle; AVG.—average; Diff.—difference.

**Table 7:** Statistical differences between periodontal pocket depths before (B) and after (A) treatment in the study (G1) and control (G2) groups.

G1	Variable	Average B	SD B±	Variable	Average A	SD A ±	St-p	Wilc. p
1	BUCC. DIS. 1	6.5	2.59	BUCC. DIS. 2	4.25	0.76	0.046	0.028
2	BUCC. MID. 1	6	1.1	BUCC. MID. 2	4	2.12	0.013	0.043
3	BUCC. MES. 1	6.33	2.07	BUCC. MES. 2	4.17	1.44	0.002	0.028
4	LING. DIS. 1	5.67	1.63	LING. DIS. 2	4.08	1.28	0.005	0.028
5	LING. MID. 1	5	0.63	LING. MID. 2	3.67	0.88	0.021	0.028
6	LING. MES. 1	7.25	3.31	LING. MES. 2	5.17	2.40	0.016	0.028
**G2**	**Variable**	**Average B**	**SD B±**	**Variable**	**Average A**	**SD A±**	**St-p**	**Wilc.p**
1	BUCC. DIS. 1	6.58	2.97	BUCC. DIS. 2	6.25	2.49	0.235	
2	BUCC. MID. 1	5.67	1.66	BUCC. MID. 2	5.58	1.66	0.363	
3	BUCC. MES. 1	6.42	0.66	BUCC. MES. 2	6.17	0.75	0.076	0.109
4	LING. DIS. 1	5.83	1.25	LING. DIS. 2	5.92	1.07	0.872	0.500
5	LING. MID. 1	5.17	0.75	LING. MID. 2	4.92	0.8	0.203	
6	LING. MES. 1	6.50	1.1	LING. MES. 2	6.08	1.07	0.042	0.068

Bucc.—buccal; Ling.—lingual; Mes.—mesial; Dis.—distal; Mid.—middle; SD—standard deviation; St-p—Student; Wilc.p—Wilcoxon.

**Table 8:** Periotest measurements before (B) and after (A) treatment in the study group (G1).

Patient/Periotest Measurements	Before Treatment	After Treatment	Average Mobility B	Max Mobility B	Average Mobility A	Max Mobility A
1	+5	+3	14.08	22	7.87	12.4
2	+3.9	+3.6				
3	+20	+10				
4	+22	+12.4				
5	+15.6	+8.6				
6	+18	+9.6				

**Table 9:** Periotest measurements before (B) and after (A) treatment in the control group (G2).

Patient/Periotest Measurements	Before Treatment	After Treatment	Average Mobility B	Max Mobility B	Average Mobility A	Max Mobility A
1	+15	+10	14.77	23	11.42	18
2	+6	+5				
3	+15	+14				
4	+23	+18				
5	+13.6	+11				
6	+16	+10.5				

**Table 10:** Comparison of Periotest measurements in the study (G1) and control (G2) groups after treatment using the Mann−Whitney test.

Group	Amount of Teeth	Average	SD	M-W *p*
1	6	7.87	3.76	
2	6	11.42	4.34	0.092

M-W—Mann−Whitney test; SD—standard deviation.

**Table 11:** Changes of bone volume (mm³) in study group (G1) before (B) and after (A) treatment.

Number of Tooth	36	36	37	36	36	27	Average Volume B/A (mm³)
Volume before treatment (mm³)	654.4	650	498	300	220	205	
Volume after treatment (mm³)	309	260	220	140	110	115	421.23/192.3
Change in percent (%)	52.8	60	65	53	50	44	

**Table 12:** Changes of bone volume (mm³) in control group (G2) before (B) and after (A) treatment.

Number of Tooth	46	47	36	37	16	27	Average Volume B/A (mm³)
Volume before treatment (mm³)	287.3	450	650	700	300	520	
Volume after treatment (mm³)	207.1	310	400	580	245	360	484.55/350.4
Change in percent (%)	28	31	38	17	18	30.7	

**Table 13:** Comparison of bone loss (mm³) after treatment in the study (G1) and control (G2) groups using Student’s *t*-test and Mann−Whitney test.

Group	Amount of Teeth	Average	SD	ST *p*	M-W *p*
1	6	192.3	83.0		
2	6	350.4	133.1	0.033	0.037

M-W—Mann−Whitney test; SD—standard deviation; ST—Student’s *t*-test.

## Data Availability

Data are available upon request.
